# Stigma, explanatory models and unmet needs of caregivers of children with developmental disorders in a low-income African country: a cross-sectional facility-based survey

**DOI:** 10.1186/s12913-016-1383-9

**Published:** 2016-04-27

**Authors:** Dejene Tilahun, Charlotte Hanlon, Abebaw Fekadu, Bethlehem Tekola, Yonas Baheretibeb, Rosa A. Hoekstra

**Affiliations:** Addis Ababa University, College of Health Sciences, School of Medicine, Department of Psychiatry, Addis Ababa, Ethiopia; King’s College London, Institute of Psychiatry, Psychology and Neuroscience, Health Service and Population Research Department, Centre for Global Mental Health, London, UK; King’s College London, Institute of Psychiatry, Psychology and Neuroscience, Department of Psychological Medicine, Centre for Affective Disorders, London, UK; The Open University, Department of Life, Health and Chemical Sciences, Milton Keynes, UK; Jimma University, College of Public Health and Medical Science, Department of Health Education and Behavioural Sciences, Jimma, Ethiopia

**Keywords:** Stigma, Developmental disorder, Autism Spectrum Disorder, Intellectual disability, Developing countries, Africa

## Abstract

**Background:**

Understanding the perspectives of caregivers of children with developmental disorders living in low-income countries is important to inform intervention programmes. The purpose of this study was to examine the stigma experiences, explanatory models, unmet needs, preferred interventions and coping mechanisms of caregivers of children with developmental disorders in Ethiopia.

**Methods:**

Participants comprised caregivers (*n* = 102) of children with developmental disorders attending two child mental health clinics in Addis Ababa. The majority (66.7 %; *n* = 68) had a diagnosis of intellectual disability (ID); 34 children (33.3 %) had autism spectrum disorder (ASD) as their primary diagnosis. All caregivers were administered a structured questionnaire via a face-to-face interview, which included an adaptation of the Family Interview Schedule, closed questions about socio-demographic characteristics, explanatory models of illness, type of interventions used or desired and coping strategies, and an open ended question regarding the family’s unmet needs.

**Results:**

Most caregivers reported experience of stigma: 43.1 % worried about being treated differently, 45.1 % felt ashamed about their child’s condition and 26.7 % made an effort to keep their child’s condition secret. Stigma did not depend on the type of developmental disorder, the child’s age or gender, or on the age or level of education of the caregiver (all *p* > 0.05). Reported stigma was significantly higher in caregivers who had sought traditional help (*p* < 0.01), provided supernatural explanations for their child’s condition (*p* = .02) and in caregivers of Orthodox Christian faith (*p* = .03). Caregivers gave a mixture of biomedical explanations (e.g. head injury (30.4 %) or birth complications (25.5 %)) and supernatural explanations (e.g. spirit possession (40.2 %) or sinful act (27.5 %)) for their child’s condition. The biggest reported unmet need was educational provision for their child (74.5 %), followed by treatment by a health professional (47.1 %), financial support (30.4 %) and expert help to support their child’s development (27.5 %). Most caregivers reported that talking to health professionals (86.3 %) and family (85.3 %) helped them to cope. Many caregivers also used support from friends (76.5 %) and prayer (57.8 %) as coping mechanisms.

**Conclusions:**

This study highlights the stigma experienced by families caring for a child with a developmental disorder. Designing interventions appropriate for low-income settings that improve awareness about developmental disorders, decrease stigma, improve access to appropriate education and strengthen caregivers’ support are needed.

**Electronic supplementary material:**

The online version of this article (doi:10.1186/s12913-016-1383-9) contains supplementary material, which is available to authorized users.

## Background

Epidemiological studies in low- and middle-income countries of the prevalence of child mental health problems, including developmental disorders, indicate that these conditions are at least as prevalent as in high-income countries [1]. In a meta-analysis, the prevalence of intellectual disability (ID) was higher in low-income countries (1.6 %) than in high-income countries (1.0 %) [2]. The global prevalence of autism spectrum disorder (ASD) is estimated to be around 0.6 %, but there are no prevalence data available from anywhere in Africa [3]. ASD and ID are lifelong developmental disorders that have a large impact not only on the individual but also on the caregivers [4]. The challenges experienced by caregivers of children with ASD or ID have been relatively well documented in high-income countries [5–8], but less is known about the experiences of caregivers from low- and middle-income countries [9–12].

Studies from high-income [5–8] and low- and middle-income countries [9, 11] have shown that families with children with autism or ID frequently experience stigma. Both felt stigma (referring to feelings of shame and the anticipation of prejudice that prevents people from talking about their experiences and impedes them looking for help) and enacted stigma (external stigma, such as evasion and discrimination) are common [13]. In India, caregivers indicated that they would be ashamed if people knew someone in their family had been diagnosed with autism [11]. In Tanzania, caregivers of children with mental illness, including ID and ASD, reported that they experienced social exclusion and discrimination [9]. Another challenge identified in previous studies is that caregivers may hold misconceptions about the perceived causes and prognosis of developmental disorder in their children [14]. Severe mental disorders [15] and autism [16] are frequently attributed to supernatural or traditional forces in sub-Saharan Africa [15] and in Asia [14]; these forces may include lineage curses, enemies, an action of the devil, or a punishment from God [15, 16].

Studies in high-income countries [8, 14, 17], India [11] and among Indian parents living abroad [18] have found that caregivers of a child with a developmental disorder tend to utilise a wide variety of treatment strategies, including biomedical as well as traditional or alternative and complementary treatments. Many caregivers also report financial difficulties, either directly, due to the costs of the treatment sought [9, 14], or indirectly, because of caregivers foregoing opportunities to engage in income generating activities [9]. In studies from high-income [8, 14] and low- and middle-income countries [9, 11, 19] caregivers have been reported to respond actively to the challenges they faced through a range of approaches: talking to a health professional, family members or religious leaders, praying and employing spiritual practices and accessing instrumental social support.

Although these previous studies have provided important insights, the challenges experienced by caregivers of children with ASD and ID have not been fully understood or described in different socio-cultural contexts. Studies in sub-Saharan Africa focusing on developmental disorders are especially rare. A study from Nigeria investigated healthcare worker perspectives on autism [16] and a qualitative study from Kenya explored the perceptions of both professionals and parents [20]. Three further studies from Africa have explored parental views [9, 21] or views from a general population sample [15] in relation to general mental health problems rather than developmental disorders specifically. Moreover, many of the previous studies in low- and middle-income countries were purely qualitative and were thus unable to compare groups statistically [9, 11, 13].

In Ethiopia, as in many other low-income countries, there are limited services for children with developmental disorders and their caregivers [12]. Out of a population of over 90 million people, nearly half of whom are children, there are only two trained child psychiatrists and the available specialised child mental health clinics are limited to the capital city, Addis Ababa. In planning future intervention programmes for children with developmental disorders it is essential to better understand the perspective of the caregivers. The aim of the present study was to describe the experiences and challenges of caring for children with ASD and/or ID among caregivers, particularly in terms of examining stigma experienced by families, understanding the perceived causes of the illness, mapping out the interventions tried and coping strategies practiced as well as determining the unmet needs of caregivers. A good understanding of these factors is essential for designing and implementing future interventions for Ethiopia and other low resource settings to improve the lives of children with developmental disorders and their families.

## Methods

### Setting

The study was conducted at the child mental health clinics at Yekatit 12 Hospital Medical College (“Yekatit 12 Hospital”) and St. Paul’s Specialised Hospital Millennium Medical College (“St Paul’s Hospital”) in Addis Ababa, the capital city of Ethiopia. Yekatit 12 and St. Paul’s hospitals are the only two public referral hospitals in Ethiopia with specialist expertise in child developmental disorders. Both hospitals provide out-patient child mental services for children with ASD and/or ID, including diagnostic assessment, medication where appropriate, signposting to available community services and ongoing follow-up.

### Study design

A cross-sectional facility-based study was carried out using a structured questionnaire administered in a face-to-face interview to caregivers of children with ASD and/or ID.

### Participants

The study population consisted of all caregivers of children under 18 years of age with a clinical diagnosis of ASD and/or ID, attending the child mental health clinics of each hospital over a period of four months. Registration records were reviewed each day to select participants who were eligible for the study. Caregivers were excluded from being invited to take part if the child was acutely disturbed or in need of emergency medical intervention. Consecutive attendees of the child mental health clinics caring for a child diagnosed with ASD and/or ID were approached to participate; all invited caregivers (*n* = 102) agreed to take part (participation rate 100 %). Diagnoses were made by a child psychiatrist and/or psychiatrist trainees following DSM-IV criteria, after a clinical observation and an interview with the child’s caregiver. The medical case notes of the child of each participating caregiver were consulted to determine the primary diagnosis of the child (ID or ASD).

### Measures

The structured questionnaire comprised five parts: socio-demographic characteristics, family experience of stigma, explanatory model of illness, type of intervention used or desired and caregiver coping strategies. The socio-demographic section collected information on the age, marital status, religion, ethnicity, and education status of the respondent. The family’s experience of stigma in the community was measured using an adapted version of the Family Interview Schedule (FIS) [22]. The FIS includes 14 questions about the family’s experience of stigma in the community. The original version of the FIS was developed for relatives of people with schizophrenia and was therefore adapted for use in this study to focus on caregivers of children with developmental disorders. An adapted version of the FIS has previously been used in Ethiopia to assess stigma in relatives of individuals with schizophrenia or major affective disorder [23]. In keeping with the version used in that study (and in contrast to the original FIS, which used a visual analogue scale), each FIS question in our survey was rated on a four-point scale where experiencing stigma in the community ‘a lot’ was given a score of 3, ‘often’ a score of 2, ‘sometimes’ a score of 1, and ‘not at all’ a score of 0. To assess the distribution of responses between groups, a total score was computed by summing the item scores, with a minimum score of 0 and a maximum score of 42. The internal consistency of this adapted FIS scale was good (Cronbach's Alpha = 0.92). The other sections of the fully structured questionnaire contained questions concerning explanatory models of illness, the perceived severity and prognosis of their child’s condition, interventions tried, coping strategies used and questions about service utilisation. The answer categories included in these closed questions were based on previous international autism studies as well as previous mental health studies in Ethiopia, and tested prior to data collection in a pre-test (please see below for more information). Each closed question also included the answer category ‘other’ (with free text specification) to allow for any answers not fitting in the pre-specified answer categories. Lastly, the type of support most needed to help improve the child’s condition was explored through an open-ended question: ‘To help your child with slow development to improve, what would help the most?’

### Data collection

The questionnaire was prepared in English, translated into Amharic and then back-translated into English to ensure consistency. The instrument was pre-tested by the first author in caregivers of children with ASD and/or ID in attendance at the child mental health clinic at Yekatit 12 Hospital and also in a group of caregivers of children attending the Nehemia Autism Centre, a centre for children with ASD in Addis Ababa. A final version of the questionnaire was established following feedback from the pre-test. Psychiatric nurses were trained to administer the questionnaire by conducting face-to-face interviews with respondents. Training was given over two days to ensure that the psychiatric nurses were familiar with the data collection procedures, the questionnaire, information sheets and consent forms. All completed questionnaires were checked for completeness, accuracy, clarity and consistency by the first author.

### Data management

Double data entry using Epidata version 3.1 [24]was employed to reduce the risk of data errors. The data were then exported to SPSS version 20 (IBM SPSS Statistics 20) for analysis.

### Data analysis

Responses to the open-ended question on support most needed were grouped following broad answer categories. The frequency distribution of all closed-ended variables was examined to check for any outliers and to see the overall distribution. The FIS total score was found to be normally distributed, permitting subsequent parametric analyses. Using stepwise multiple linear regression it was tested whether any of the demographic, clinical or explanatory model and service use related variables could predict FIS sum scores. Non-parametric Mann–Whitney tests were used to examine whether scores on individual stigma items were related to the type of developmental disorder (ID vs ASD). Results were interpreted as significant when *p* < 0.05.

### Ethical considerations

Ethical approval was obtained from the Institutional Review Board of the College of Health Sciences of Addis Ababa University and the Human Research Ethics Committee of the Open University (UK). Authorisation from both child mental health clinics was obtained. All study participants were informed about the purpose of the study and written informed consent was secured from all participants prior to the start of data collection.

## Results

### Demographic characteristics

Data were available for 102 participants. The mean age of the respondents was 36.9 years (SD = 8.9); the majority were Orthodox Christians (*n* = 73; 71.6 %), married (*n* = 70; 68.6 %), were urban residents (*n* = 82; 80.4 %) and housewives (*n* = 49; 48.0 %). About a fifth of the respondents (*n* = 22; 21.6 %) were uneducated; 60.8 % (*n* = 62) had received at least some formal education (ranging from any primary (grade 1-8) to any secondary school education (grade 9-12); the remaining caregivers (*n* = 18; 17.6 %) had completed a form of tertiary education. See Table 1. Among the cases, 68 children (66.7 %) had a diagnosis of ID and 34 children (33.3 %) had ASD as their primary diagnosis. The medical case notes of 10 out of 34 children with ASD explicitly mentioned that they had co-comorbid ID, but this is likely to be an underestimate of co-morbidity as high functioning cases of ASD are rarely diagnosed in Ethiopia. The mean age of children with developmental disorder was 8.2 ± 3.3 years. Most of the children (*n* = 77; 75.5 %) were male. Of the 102 caregivers of children with developmental disorder, five (4.9 %) had one or more other children with developmental problems (Table 1).

**Table 1:** Demographic and clinical characteristics of the respondents and association with scores on the adapted Family Interview Schedule (FIS) stigma scale

	Characteristic	*N* (%)	Mean FIS score	Standardised B coefficient	t-value	*p*-value
Caregiver characteristics						
Age (years)	Mean age 36.9 years					
	<25	9 (8.8)	14.9			
	25-34	30 (29.4)	10.5			
	35-44	41 (40.2)	12.8			
	≥45	22 (21.6)	12.2			
Religion	Orthodox	73 (71.6)	13.9	.21	2.23	.028
	Protestant	16 (15.7)	6.6			
	Muslim	13 (12.7)	9.7			
Marital status	Married	70 (68.6)	11.0			
	Never married	9 (8.8)	11.7			
	Formerly married	12 (11.8)	18.3			
	Not applicable	11 (10.8)	13.5			
Level of education	No formal education	22 (21.6)	13.8			
	Completed grade (1-12)	62 (60.8)	11.6			
	Diploma and above	18 (17.6)	8.4			
Occupation	Farmer	11 (10.8)	9.9			
	Housewife	49 (48.0)	13.2			
	Employed	34 (33.3)	10.7			
	Unemployed	8 (7.8)	13.9			
Residence	Urban	82 (80.4)	12.4			
	Rural	20 (19.6)	11.6			
Child characteristics						
Age of child with developmental disorder	Mean age 8.2 years					
	1-6 years	32 (31.4)	11.4			
	7-12 years	61 (59.8)	11.8			
	13-18 years	9 (8.8)	17.6			
Gender	Boy	77 (75.5)	11.5			
	Girl	25 (24.5)	14.4			
Siblings with developmental problems	No	97 (95.1)	12.3			
	Yes	5 (4.9)	10.8			
Type of developmental disorder	Autism spectrum disorder	34 (33.3)	11.9			
	Intellectual disability	68 (66.7)	12.3			
Caregiver explanatory models						
Supernatural causal explanation	No	46 (45.1)	9.2			
	Yes	56 (54.9)	14.6	.22	2.40	.018
Biomedical causal explanation	No	41 (40.2)	10.2			
	Yes	61 (59.8)	13.6			
First looked for help	Traditional institution	56 (54.9)	10.7			
	Biomedical institution	46 (45.1)	14.0			
Ever sought help from traditional institution	No	34 (33.3)	7.0			
	Yes	68 (66.7)	14.8	.27	2.95	.004
Traditional treatment tried	No	42 (41.2)	8.8			
	Yes	60 (58.8)	14.6			
Biomedical treatment tried	No	61 (59.8)	12.4			
	Yes	41 (40.2)	11.9			

Note: *FIS* Family Interview Schedule; the original FIS was adapted to be appropriate for caregivers of children with developmental disorders

### Experienced stigma and explanatory models

**Fig. 1:**
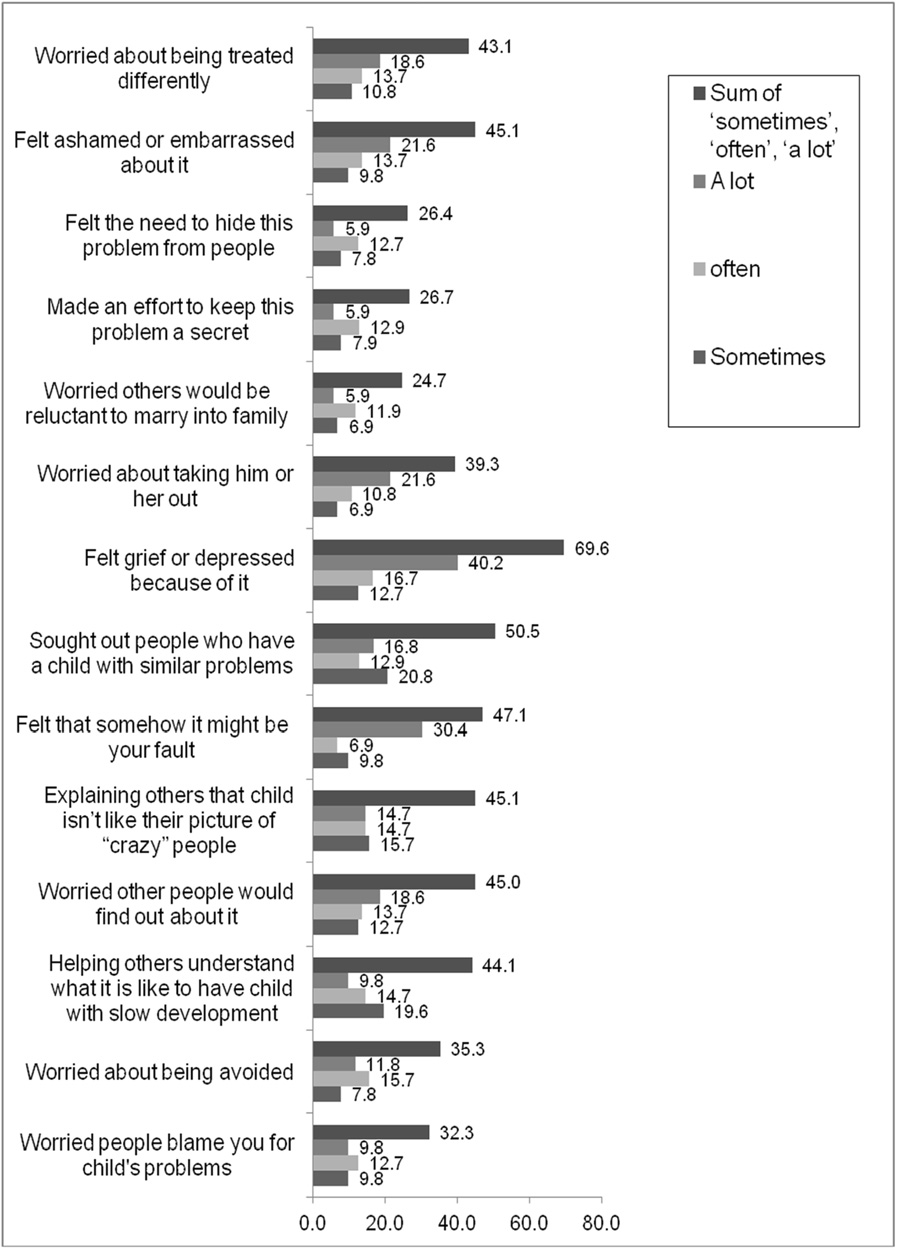
Pattern of positive responses on the Family Interview Schedule questionnaire in caregivers of children with developmental disorders

Caregivers endorsed experience of stigma on many items of the adapted FIS (see Fig. 1). For example, 44 out of 102 participants (43.1 %) indicated they worried ‘sometimes’ ‘often’ or ‘a lot’ about being treated differently. Likewise, many felt ashamed or embarrassed about their child’s condition (*n* = 46; 45.1 %), felt a need to hide the problem from people in the community (*n* = 27; 26.4 %), or made an effort to keep their child’s condition a secret (*n* = 27 out of 101 responses; 26.7 %), worried that people would be reluctant to marry into their family (*n* = 25; 24.7 %) and worried about taking their child out of the house (*n* = 40; 39.3 %). Other experiences of caregivers included feeling depressed about their child’s condition (*n* = 71; 69.6 %), seeking caregivers of a child with similar problems (*n* = 51 out of 101 responses; 50.5 %), feeling that their child’s problem is their fault (*n* = 48; 47.1 %) and explaining to others that their child does not fit typical cultural stereotypes of mentally ill individuals (*n* = 46; 45.1 %) (Fig. 1).

Caregivers cited a mixture of biological and supernatural factors as causes for their child’s condition (Table 2). The most common biomedical explanations were head injury (*n* = 31; 30.4 %), birth complications (*n* = 26; 25.5 %), epilepsy (*n* = 21; 20.6 %), pathogens (*n* = 12; 11.8 %) and a family history (*n* = 12; 11.8 %). Frequently cited supernatural explanations included spirit possession (a spirit taking control over one’s thinking and actions; *n* = 41; 40.2 %), a sinful act (a direct result of the caregiver’s transgression *n* = 28; 27.5 %), punishment from God (specific attribution of the consequence of a sinful act to punishment from God *n* = 26; 25.5 %), evil eye or “*buda”* (a spell cast by the eye, inflicting injury or misfortune on the person being looked at; *n* = 20; 19.6 %), and curse or bewitchment (harm inflicted by magical acts or supernatural powers instigated by another person or by supernatural beings; *n* = 10; 9.8 %). Over half (*n* = 56; 54.9 %) of respondents gave at least one supernatural causal explanation, while 59.8 % (*n* = 61) gave at least one biomedical explanation. Biomedical and supernatural causal explanations were not mutually exclusive, with 37 participants (36.3 %) providing both biomedical and supernatural explanations.

**Table 2:** Caregiver perceived causes of developmental disorder in the child^a^

Perceived causes of child developmental disorder	Number	Percent
Spirit possession	41	40.2
Sinful act	28	27.5
Punishment from God	26	25.5
Evil eye	20	19.6
Curse/bewitchment	10	9.8
Head injury	31	30.4
Birth complications	26	25.5
Epilepsy	21	20.6
Pathogen/infectious cause	12	11.8
Family history	12	11.8
Drinking alcohol in pregnancy	3	2.9
Chewing *Catha edulis* (khat) in pregnancy	2	2.0

^a^Respondents could provide multiple answers

Even though the majority (*n* = 69; 67.6 %) of participants believed that their child’s developmental problems were very or quite severe, most of the respondents (*n* = 94; 92.2 %) believed that their child’s problems could be cured. Only a minority of respondents thought that their child’s condition could be transmitted to other people (*n* = 7; 6.9 %). A slightly higher number (*n* = 12, 11.8 %) indicated that other people thought their child’s condition was contagious (Table 3).

**Table 3:** Caregiver perceptions about the child with a developmental disorder

Variable	Number	Percent
Believe that child’s problems with autism/ID can be cured		
Yes, cured	94	92.2
Not cured, but improved	8	7.8
No cure or improvement	0	0
How severe do you think your child’s problem with developing slowly is?		
Very severe	44	43.1
Quite severe	25	24.5
Not too severe	25	24.5
Not severe at all	8	7.8
Do you think your child’s condition can be transmitted to other people?		
No	95	93.1
Yes	7	6.9
Do you think other people think that your child’s condition can be transmitted to other people?		
No	90	88.2
Yes	12	11.8

### Traditional and biomedical support sought

When asked where they went to seek help for their child, more than half of the caregivers indicated they first sought help from traditional places (*n* = 56; 54.9 %), while just under half of the participants first approached a biomedical institution (*n* = 46; 45.1 %). When asked what other types of help they had sought prior to coming to the current child mental health clinic, the majority had attended a hospital (*n* = 83; 81.4 %) and/or a private clinic (*n* = 27; 26.5 %), and many had visited traditional institutions, including centres for religious healing (e.g. holy water (*n* = 53; 52.0 %), a church or priest (*n* = 35; 34.3 %)) or different types of traditional healers (Table 4).

**Table 4:** Help-seeking by caregivers of a child with developmental disorder

Help-seeking	Number	Percent
First place where help sought		
Biomedical (modern health institution)	46	45.1
Traditional	56	54.9
Have you ever looked for help from any of the following^a^		
Holy water	53	52.0
Church/priest	35	34.3
*Debtera* (spiritual healers in Orthodox Christian clergy)	7	6.9
Herbalist	11	10.8
Mosque	9	8.8
*Kalicha* (spiritual healers in Muslim clergy)	6	5.9
*Tanquaye* (Sorcerer)	5	4.9
*Wogesha* (traditional physical therapy including massage and bone setting)	5	4.9
Hospital	83	81.4
Private clinic	27	26.5
Public health centre	22	21.6
Health extension workers	6	5.9
Private pharmacy	6	5.9
Broad classification of help sought		
Ever sought traditional help	68	66.7

Note: ^a^ Respondents could provide multiple answers

### Type of interventions tried

Caregivers reported using both biomedical treatments (tablets (*n* = 40; 39.2 %) or injections (*n* = 4; 3.9 %) received through a health facility) and traditional interventions (prayer (*n* = 48; 47.1 %), *kitab* (a written script tied on the arm or neck; *n* = 8; 7.8 %), slaughtering a sheep (*n* = 4; 3.9 %), or fumigating (making excessive use of smoke by burning incense; *n* = 2; 2.0 %)) as treatment interventions. A subgroup of caregivers also indicated they had used beating (*n* = 19; 18.6 %) or chaining (*n* = 9; 8.8 %) to manage their child (Additional file 1: Table S1). Many (*n* = 27, 26.5 %) caregivers had tried both traditional and biomedical treatment for their child.

Table 1 summarises the results of the analyses examining the association of demographic and clinical characteristics with the sum score of the adapted FIS, using stepwise multiple regression. The experience of stigma, as indicated by the FIS sum score, was associated with caregivers providing a supernatural causal explanation (*p* = 0.02), but not associated with a biomedical causal explanation (*p* > 0.05). Moreover, experienced stigma was significantly higher in caregivers of Orthodox Christian faith (*p* = .03) and in caregivers who had sought help from traditional institutions (*p* < 0.01). Having included ‘help sought from traditional institution’ in the regression model, the variable ‘tried traditional treatments’ did not contribute significant additional predictive information (*p* > .05). Likewise, whether caregivers had ever tried biomedical treatments and whether families first looked for help from a biomedical or traditional institution was not associated with the FIS score (both *p* > .05). The FIS total score also did not depend on the type of developmental disorder (ID vs. ASD), the child’s age or gender, the age or level of education of the caregiver or whether the family lived in a rural or urban area (all *p* > 0.05). Finally, when considering each stigma item in isolation, no difference in reported stigma was found in relation to type of developmental disorder (all *p* > 0.05).

### Unmet need

The most common unmet needs expressed by caregivers were appropriate educational provision for their child (*n* = 76; 74.5 %), treatment by a health professional (*n* = 48; 47.1), financial support (for instance to buy food) (*n* = 31; 30.4 %), access to support from professionals in managing their child and supporting their child’s skills development (*n* = 28; 27.5 %) and access to expert information and advice about their child’s condition (*n* = 23; 22.5 %) (Additional file 1: Table S1). All responses concerned desired support from outside rather than within the family. The phrasing of the question (‘To help your child with slow development to improve, what would help the most?’), with its focus on support to *improve* the child’s development, is likely to have contributed to respondents referring to external support.

### Coping strategies

Various coping mechanisms, including talking to someone, seeking religious help and using substances/drugs, were used to deal with emotional difficulties arising as a result of caring for a child with ASD or ID. Caregivers most often spoke to a health professional (*n* = 88; 86.3 %), talked to family members (*n* = 87; 85.3 %) or talked to friends (*n* = 78; 76.5 %), and also often used prayer (*n* = 59; 57.8 %) as a coping strategy. Adverse coping strategies such as chewing *Catha edulis* (khat) (*n* = 5; 4.9 %), drinking alcohol (*n* = 4; 3.9 %) and smoking cigarettes (*n* = 3; 2.9 %) were used by a minority of respondents (Additional file 1: Table S1).

### Gateway to the clinic

**Fig. 2:**
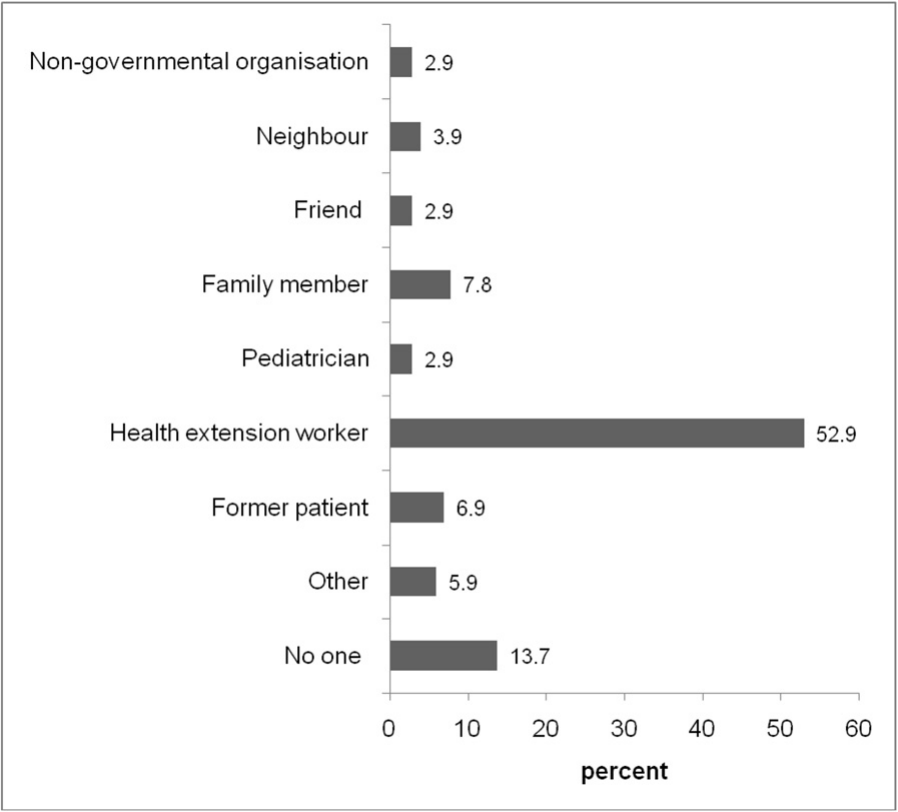
Source of information leading to attendance of caregiver at child mental health clinic

The most common source of information that had led the family to attend the child mental health clinic was a community-based health extension worker, with 52.9 % of caregivers citing their help in finding the way to the clinic (Fig. 2).

## Discussion

### High levels of stigma experienced by caregivers

This study indicates that stigma experienced by caregivers, including worrying about being treated differently, feeling ashamed or embarrassed about their child’s condition, making an effort to keep their child’s condition a secret and explaining to others that their child does not fit stereotypical conceptions of mental illness or ‘madness’, are common among caregivers of children with ASD or ID. Other experiences, such as feeling depressed, seeking out other people caring for a child with similar problems, or feeling that the problem is their fault are also common. This finding is consistent with research conducted in high-income countries that indicated the majority of caregivers experienced felt stigma [5–8, 14]. Our study findings were also in accordance with other studies conducted in low- and middle-income countries, namely that caregivers of children with developmental disorders often experienced embarrassment, shame and guilt [9, 11, 13, 23]. In a previous study in Ethiopia that assessed stigma using the FIS in relatives of people with schizophrenia or major affective disorder found similarly high levels of stigma [23]. Investigators from India, Tanzania and China reported that the majority of caregivers of children with developmental disorders felt tired, depressed, exhausted and worried about their child’s future role in society [9, 11, 19]. However, these studies were purely qualitative and were unable to examine quantitatively which factors may be associated with stigma. Our findings suggest that caregivers seeking help from traditional institutions experience significantly higher levels of stigma than caregivers who did not seek this type of support. Moreover, Orthodox Christian religion and providing a supernatural explanation for the child’s problems were predictive of high reported stigma. As this is a cross-sectional study, it was not possible to examine the direction of causation of the association between traditional support and stigma experienced. That is, it remains unclear whether engaging with traditional institutions may increase experienced stigma, or whether families who experience high levels of stigma are more inclined to seek support from traditional institutions.

### Explanatory model of illness for caregivers

In this study, caregivers cited a mix of biomedical and supernatural factors as causes for their child’s condition. Many caregivers reported that they perceived both supernatural factors, such as spirit possession, the result of a sinful act or punishment from God, and biomedical factors, such as a head injury, birth complications, or epilepsy to be the cause of their child’s developmental disorder. This result is consistent with studies in Taiwanese parents [14] and Indian parents living abroad [18], who also reported a mix of biomedical and supernatural causes of their child’s condition**.** Similarly, in a previous study from Ethiopia, where the explanatory models of parents of in-school children for child mental disorders were investigated, the majority endorsed both biomedical and supernatural courses [21]. These results suggest that supernatural and biomedical belief systems often co-exist. It should be noted that, given that all study participants were recruited from a specialist referral hospital, selection bias may have led to a higher proportion of participants from our study endorsing biomedical causes than may be observed in the general Ethiopian population.

Most respondents (92.2 %) in this study believed the child’s condition to be curable. This finding suggests caregivers may have somewhat unrealistic expectations of what can be achieved with interventions or treatment, perhaps suggesting a risk for dissatisfaction among caregivers with the outcome for future interventions. However, ‘social desirability’ bias may also have played a role: since the study was conducted in a mental health clinic and the data collectors were psychiatric nurses, caregivers may have been inclined to over emphasise their belief in a (biomedical) cure. A further explanation for the strong confidence in curability could be religious beliefs. A study carried out among South Asian Muslim immigrant families reported that families understood the task of raising a child with autism in religious terms [25]. In that study, parents reported that keeping to the precepts of Islam would help them to raise their developmentally disabled children as normally as possible. The high level of belief in curability in our study may similarly be explained by the fact that Ethiopia is a deeply religious society. Reflective of Ethiopian society as a whole, all our participants were religious and not believing in a cure could be understood as questioning the power of God. In the Kenyan study, parents of children with ASD reported that they hoped for a cure and sought treatment with this expectation in mind [20].

The majority (67.6 %) of the participants in this study indicated that they believed their child’s developmental disorder to be very or quite severe. In the Ethiopian context, where access to child mental health services is extremely limited, only the more severe cases will reach the clinic. Therefore our sample is likely to include more severe cases of developmental disorders than the full ID and ASD spectrum that may attend clinics in better resourced countries. As there are no validated tests of intellectual ability for use in Ethiopia it was impossible to provide a quantitative description of the extent of childrens’ developmental disability.

### Interventions tried by caregivers

Many caregivers indicated they used a combination of traditional and biomedical interventions to help their child. This is comparable to studies done in high-income countries [8, 14] that reported caregivers used multiple services and treatment strategies to help their developmentally disabled child including psychological, medical and paramedical services [8], as well as special diets and traditional treatments [14, 18]. For example in Taiwan parents reported engaging in spiritual rituals such as paying a monk to read the Buddhist Bible and changing the name of the child to change his “fate”. Similarly, our study found rituals related to the prevalent religions in Ethiopia (Orthodox and protestant Christianity and Islam) and traditional belief systems to be common. The wide range of interventions tried by caregivers may reflect the fact ASD and ID are lifelong conditions, so caregivers keep looking for answers and treatments.

A considerable minority of caregivers indicated they have beaten (18.6 %) or chained (8.8 %) their child to manage their child’s problems. Beating a child in an attempt to teach them to improve their behaviour is still common in Ethiopia. However, in a study in which we interviewed service providers for children with autism [12] it was highlighted that chaining is usually done not as a punishment, but to protect the child from harming themselves or others when there is no-one to look after the child. Interventions are urgently needed to support families in the community and to discourage harmful practices such as beating and chaining.

### Unmet needs of caregivers

The majority of caregivers (74.5 %) indicated an unmet need for appropriate educational provision for their child, followed by treatment by a health professional (47.1 %). In addition many caregivers expressed they needed financial support (30.4 %), access to professionals to help support their child’s behaviour and skills development (27.5 %) and access to expert information and advice about their child’s condition (22.5 %). These perceived needs of caregivers in our study are comparable to studies from high-income countries [8, 10], in which the majority expressed a need for educational services, health service provision, support for social activities, and access to information. A systematic review of studies conducted in Brazil indicated a similar set of unmet needs [26]. Likewise, a study conducted in Tanzania [9] indicated that caregivers expressed a desire for support with schooling, professional assistance, health education and information, and social supports. This common set of unmet needs reported by caregivers among different studies is likely to be because of the similarity of the behaviours or needs of children with ASD or ID regardless of the setting they live in. However, the service provision for children with developmental disorder in high-income countries is rather different from the provision available in most low- and middle-income countries. Even though the provision may be perceived as inadequate most children in high-income countries will get some type of support and special education (although stark differences may exist between socioeconomic groups). In contrast, in Ethiopia most children with developmental disorders do not get any provision; most remain undiagnosed and specialist schooling and intervention is unavailable in rural areas, where 85 % of the population lives [12]. Although the service provision for children with developmental disorders in low- and middle-income countries is not well-documented, the challenges reported in Ethiopia are likely to also apply to other low resource countries and settings.

### Coping strategies for caregivers

The most common coping mechanisms used by caregivers in our study were talking to health professionals, talking with family or friends and prayer. This result is consistent with a systematic review of coping strategies of families affected by autism in Brazil, where caregivers were assisted by access to medical care, information exchange between families and seeking religious support [26]. Talking to family and friends was another important coping mechanism reported frequently by parents from the Philippines [27] and in studies conducted in high-income countries [10, 18, 28]. Our study indicated more than half (57.8 %) of the respondents reported prayer as a coping mechanism, reflecting the importance of religious faith within society. This finding also highlights that, while seeking help from traditional institutions was related to higher experienced stigma, many caregivers find support in their religious beliefs and rituals through prayer. In a study from the Philippines, religion and spiritual practices seemed to provide positive social support for parents of developmentally disabled children; church members offered emotional support, guidance, frequent company and acceptance of their children [27]. In a study from rural Ethiopia in relatives of people with schizophrenia many relatives were also inclined towards prayer for guidance and talking with someone about their problems as a coping mechanism [29]. Similar to our findings the study participants valued their contact with health professionals in enabling them to cope. This might be a reflection of the high status of health professionals in Ethiopia and indicates an important role of the health sector in supporting caregivers of children with child developmental disorders.

### Gateway to the clinic

In the majority of cases (52.9 %) the family found their way to the clinic through referral by a health extension worker. Health extension workers provide basic health services in the local community. Since 2003 the Ethiopian government has trained 39,000 of these community health workers, with two health extension workers deployed in each community. Our study suggests that these workers play an important role in identifying developmental disorders and thus form a gateway to specialised help. As part of our HEAT+ research project we are currently evaluating the impact of brief mental health training for health extension workers in improving awareness of mental health problems and developmental disorders [30]. This training aims to improve the detection of developmental disorders in the rural areas of Ethiopia (where most health extension workers are deployed) and to help decrease stigma in the community by equipping the health worker with the skills and knowledge needed to raise mental health awareness in the local community.

### Limitations and strengths

Limitations of the study include that the respondents lived primarily in urban areas; the findings may not be generalisable to more rural settings in Ethiopia. The study was facility-based, and thus was biased towards those with higher educational level and ability to access biomedical care. The experiences of these caregivers may differ from families whose child with similar problems has not (yet) been diagnosed. We were not able to determine how long ago the caregivers were first informed of their child’s diagnosis, nor since how long the caregivers had been concerned about their child’s development. The perspectives of caregivers who have long been aware of their child’s condition may be different from those who have only recently become aware their child’s development is atypical. Also, our sample of caregivers of a child with ASD was small, providing limited power to compare the stigma associated with to that of ID only. Additionally, the cross-sectional study design restricts our ability to draw causal or temporal associations. Lastly, since the study was conducted by psychiatric nurses and touches on potentially sensitive issues, the possibility that respondents reported information based on their perception of what the interviewer wanted to hear (i.e. a ‘social desirability’ bias) cannot be excluded. However, the study is the first study in Ethiopia among the caregivers of children with ASD or ID and provides information in an area of public health of which very little is known, not just in Ethiopia, but in sub-Saharan Africa more widely. Moreover the high participation of participants and high consistency with other studies in caregivers of children with developmental disorders supports the reliability of our findings.

### Implications

This research has a number of implications for professionals working with caregivers and children with developmental disorders in Ethiopia and other low resource settings. First, in response to the high levels of felt stigma in caregivers, there is a need to design interventions to improve public awareness about developmental disorders, decrease stigma and improve access to appropriate services. Second, in response to the finding that stigma experience was significantly stronger in caregivers who had sought help from traditional institutions, there is a need to work collaboratively with traditional and faith healers, for example by organising joint public awareness and anti-stigma efforts with these traditional institutions [31]. We do not advocate discouraging seeking help from traditional and religious institutions, as many caregivers feel supported by these systems, also illustrated by the high percentage of caregivers indicating they used prayer as a coping strategy. Third, we also found that many parents hold both supernatural and biomedical causal beliefs and that the two aetiologies are not mutually exclusive, with most participants giving both biomedical and supernatural explanations. Based on this finding, it is necessary to train healthcare providers to be empathetic to the needs and conditions of caregivers and be open to the possibility of collaborative engagement with traditional healers for the holistic care of children with developmental disorders [31]. Health professionals need to respect caregivers’ beliefs and supernatural explanations, while also sensitively providing psycho- education on causes and helpful strategies to support their child, and discourage harmful practices such as beating and chaining. Fourth, the finding that caregivers’ coping mechanisms include talking to health professionals, family and friends as a main mechanism to cope the problems can help professionals to prepare different strategies in counselling and the delivery of psychosocial interventions. In low-resource settings such as Ethiopia community-based psychosocial interventions delivered by non-specialists (e.g. community health workers, primary care workers or peers) are a viable strategy to scale-up support for families with children with developmental disorders [32]. Though, since most parents believe their child can be cured, this expectation of a cure needs to be born in mind when designing a psychosocial intervention. Fifth, the finding that health extension workers form the main gateway to access to biomedical services highlights the importance of increasing awareness of developmental disorders among health extension workers to increase identification of ASD and ID in the local community.

### Conclusion

Caregivers of children with ASD or ID in Ethiopia face many challenges, including high levels of stigma and a lack of appropriate provision for their child. Reported stigma was significantly stronger in caregivers who had sought help from traditional institutions or had supernatural explanations for their child’s condition. The study has implications for policies to reduce stigma, increase awareness about the causes of developmental disorders and address the needs of caregivers of developmentally disabled children. Interventions to improve awareness about developmental disorders, to decrease stigma, and improve access to appropriate education and support for caregivers are warranted.

### Ethics approval and consent to participate

Ethical approval was obtained from the Institutional Review Board of the College of Health Sciences of Addis Ababa University and the Human Research Ethics Committee of the Open University (UK). Authorisation from both child mental health clinics was obtained. All study participants were informed about the purpose of the study and written informed consent was secured from all participants prior to the start of data collection.

### Consent for publication

Not applicable.

### Availability of data and materials

The dataset will not be shared because it is being used for an ongoing PhD thesis.

## Additional file

Additional file 1: Table S1. Type of treatment tried, unmet needs and coping mechanisms of caregivers of children with developmental disorders. (DOCX 13 kb)

## Abbreviations

ASD: autism spectrum disorder
FIS: family interview schedule
ID: intellectual disability
