# Supplementary material for: Stigma, explanatory models and unmet needs of caregivers of children with developmental disorders in a low-income African country: a cross-sectional facility-based survey
Source: BMC Health Serv Res. 2016 Apr 27;16:152. doi: 10.1186/s12913-016-1383-9 (PMC4847244; doi:10.1186/s12913-016-1383-9)
Supplement: Additional file 1: Table S1. — Type of treatment tried, unmet needs and coping mechanisms of caregivers of children with developmental disorders. (DOCX 13 kb) [file 12913_2016_1383_MOESM1_ESM.docx]

**Additional file 1**

**Table S1:** Type of treatment tried, unmet needs and coping mechanisms of caregivers of children with developmental disorders

| **Sociodemographic characteristics** | **Number** | **Percent** |
| --- | --- | --- |
| **Type of treatment tried*** |  |  |
| Tablets from health facility | 40 | 39.2 |
| Injection from health facility | 4 | 3.9 |
| Chaining | 9 | 8.8 |
| Beating | 19 | 18.6 |
| Fumigating | 2 | 2.0 |
| Prayer | 48 | 47.1 |
| Slaughtering a sheep | 4 | 3.9 |
| *Kitab* (a written script tied on the arm or neck) | 8 | 7.8 |
| **Help most needed*** |  |  |
| Educational provision for child | 76 | 74.5 |
| Treatment by health professional | 48 | 47.1 |
| Professional help to manage child and/or support child’s skills development | 28 | 27.5 |
| Financial support | 31 | 30.4 |
| Expert information and advice about child’s condition | 23 | 22.5 |
| **Help sought to cope with child’s condition*** |  |  |
| Talking to family | 87 | 85.3 |
| Talking to friends | 78 | 76.5 |
| Talking to health professional | 88 | 86.3 |
| Prayer | 59 | 57.8 |
| Drinking alcohol | 4 | 3.9 |
| Chewing khat | 5 | 4.9 |
| Smoking cigarettes | 3 | 2.9 |

* Respondents could provide multiple answers
